# Exosomes in asthma: Underappreciated contributors to the pathogenesis and novel therapeutic tools

**DOI:** 10.1002/iid3.1325

**Published:** 2024-06-27

**Authors:** Zahra Kanannejad, Samaneh Arab, Saeede Soleimanian, Amirhossein Mazare, Nasim Kheshtchin

**Affiliations:** ^1^ Allergy Research Center Shiraz University of Medical Sciences Shiraz Iran; ^2^ Department of Tissue Engineering and Applied Cell Sciences, School of Medicine Semnan University of Medical Sciences Semnan Iran; ^3^ Department of Immunology, School of Medicine Shiraz University of Medical Sciences Shiraz Iran

**Keywords:** exosome, extracellular vesicle, asthma, allergy, intercellular communication, immunotherapy

## Abstract

**Objective:**

Asthma, a chronic inflammatory disease with diverse pathomechanisms, presents challenges in developing personalized diagnostic and therapeutic approaches. This review aims to provide a comprehensive overview of the role of exosomes, small extracellular vesicles, in asthma pathophysiology and explores their potential as diagnostic biomarkers and therapeutic tools.

**Methods:**

A literature search was conducted to identify recent studies investigating the involvement of exosomes in asthma. The retrieved articles were analyzed to extract relevant information on the role of exosomes in maintaining lung microenvironment homeostasis, regulating inflammatory responses, and their diagnostic and therapeutic potential for asthma.

**Results:**

Exosomes secreted by various cell types, have emerged as crucial mediators of intercellular communication in healthy and diseased conditions. Evidence suggest that exosomes play a significant role in maintaining lung microenvironment homeostasis and contribute to asthma pathogenesis by regulating inflammatory responses. Differential exosomal content between healthy individuals and asthmatics holds promise for the development of novel asthma biomarkers. Furthermore, exosomes secreted by immune and nonimmune cells, as well as those detected in biofluids, demonstrate potential in promoting or regulating immune responses, making them attractive candidates for designing new treatment strategies for inflammatory conditions such as asthma.

**Conclusion:**

Exosomes, with their ability to modulate immune responses and deliver therapeutic cargo, offer potential as targeted therapeutic tools in asthma management. Further research and clinical trials are required to fully understand the mechanisms underlying exosome‐mediated effects and translate these findings into effective diagnostic and therapeutic strategies for asthma patients.

## Graphical abstract

Exosome in ashtma.

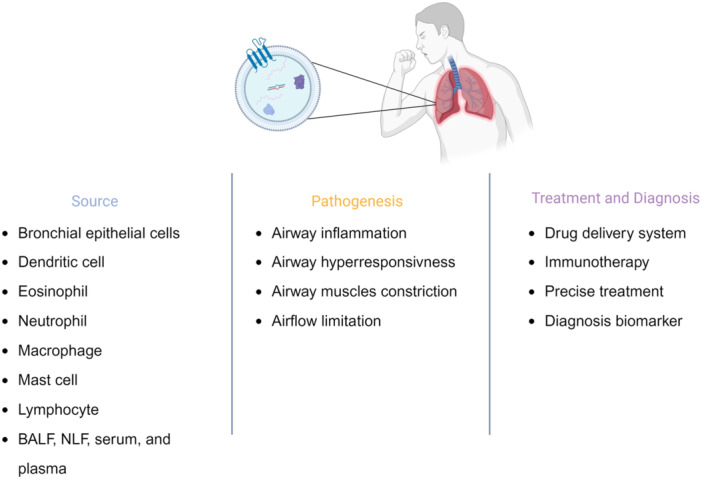

## 1 INTRODUCTION

Asthma, a chronic airway inflammation, affects approximately 300 million individuals worldwide.^1^ Asthma is characterized by airway obstruction, inflammation, and remodeling. The development of this disease is influenced by a complex interplay between genetic and environmental factors, along with the involvement of diverse immune and nonimmune cells.^2^, ^3^, ^4^ Asthma inflammation involves the interaction and secretion of soluble mediators by various cell types, including immune cells (such as mast cells, eosinophils, and T cells) as well as structural cells (such as epithelial and endothelial cells, and fibroblasts).

Exosomes have emerged as crucial mediators of intercellular communication in recent years. They facilitate the transfer of diverse molecules, including proteins, lipids, DNA, and RNA, between cells through direct contact or circulation. Exosomes can, therefore, alter the function of target cells and contribute to the development of a pathological state by its components. Many studies have investigated exosomes in association with different inflammatory conditions especially cancer,^5^, ^6^ infectious diseases,^7^ and asthma.^8^, ^9^, ^10^

Exosomes have been implicated in the pathophysiology of asthma, as well as its diagnosis and treatment. Various immune and structural cells in the lungs, such as epithelial cells, smooth muscle cells, fibroblasts, mast cells, neutrophils, eosinophils, dendritic cells, T cells, B cells, and regulatory T (Treg) cells, can release and receive exosomes that modulate their function and phenotype in asthma.^11^ This review provides an overview of the recent progress in understanding how exosomes contribute to the pathogenesis of asthma and how they can be used as biomarkers for asthma therapy.

## 2 EXTRACELLULAR VESICLES (EVS)

EVs serve as potent vehicles for cell‐cell communication and a way for transferring cell contents.^12^ EVs are classified into three main forms based on size, cargo, function, secretion pathway and biogenesis: microvesicles (MVs), exosomes, and apoptotic bodies.^13^ Various physiological conditions including immunity,^14^ regeneration of tissues,^15^ stem cell integrity,^16^ and transporting signals in central nervous system (CNS)^17^ are affected by EVs production. Clinically, EVs could also be used as innovative diagnostic and therapeutic biomarkers as well as monitoring therapeutic response in various conditions.^18^, ^19^

### 2.1 Exosomes

Exosomes, a subtype of extracellular vesicles, are released by various cells and exhibit a range of sizes, typically between 30 to approximately 200 nanometers.^20^ Exosomes originate from late endosomes or multivesicular bodies (MVBs) through the formation of intraluminal vesicles (ILVs) pathway. Alternatively, they can also be generated directly from the plasma membrane through exocytosis. Distinct cytosolic components such as various proteins and lipids are inserted into ILVs. Most of them are addressed into extracellular space via fuse with plasma membrane as exosomes. Rest of MVBs go through degradation with multiple fates mechanisms, releasing of exosome components into cytosol space, transferring to lysosomes or leaking into nucleus or endoplasmic reticulum (ER).^13^ In addition, upon recycling endosome, exosomes secret to outside of cells. The endosomal sorting complex (ESCRT) involves in the formation of MVBs, vesicle formation, and protein cargo arranging.^21^

Exosomes are heterogeneous in composition, containing a broad range of proteins such as tetraspanins (transmembrane proteins), major histocompatibility complex‐I (MHC‐I) and ‐II as antigen presenting molecules, adhesion molecules, integrin and glycoproteins.^13^ In addition to proteins, exosome contains nucleic acids, including DNA, mRNA, noncoding RNA, and microRNAs (miRNA) as well as various lipids such as phosphatidylethanolamine, phosphatidylcholine, phosphatidylinostol, phosphatidylserine, sphingomyelin ceramides and gangliosides.^22^ Some common and distinctive proteins in the exosome proteomic mixture could be utilized as exosome markers, including CD63, CD9, and CD81 (tetraspanins), heat shock protein (HSP)70, HSP90, glyceraldehyde 3‐phosphate dehydrogenase (GAPDH), nitric oxide synthase, and catalase (enzymes), endosomal origin proteins such as Tsg101 and ALIX and actin as a cytoskeleton protein. Exosome secreted miRNAs impact on regulation of gene expression, used as prominent biomarkers for various diseases and biological phenomena.^23^, ^24^

Exosomes facilitate cellular communication by transmitting signals from host cells to target cells. Internalizing of exosomes through endocytosis, communication of cells via receptor and ligands, and direct fusing of cell membranes result in consideration of exosomes as an effective tool for genetic material and functional cargo transferring.^25^ Exosome augmentation causes interaction of parental cells with proximal or distal recipient cells, leading to alteration of epigenetic profile of target cells by transfer of bioactive molecules such as RNAs, proteins, and lipids and also delivery of activating receptors to these cells.^26^ In immunity setting, exosomes participate in regulation of immune responses via intercellular communication by impacting on immune tolerance, antigen presentation, stimulation of immune response, and inhibition of immunity. Different cargos are carried by exosomes from various immune cells and they can affect how the target cell acts and works. Some of these cells cause inflammation to get rid of pathogens, so their exosomes may be involved in inflammatory diseases that damage tissues, cause autoimmunity, or trigger allergies.^10^, ^27^ On the other hand, some immune cells may help balance and control immune responses. These cells produce exosomes that may help cancer and infectious diseases to grow.^28^, ^29^

Exosomes have emerged as promising entities in both diagnostic and therapeutic applications in recent years. As diagnostic biomarkers, exosomes offer a noninvasive approach to disease detection and monitoring. These small vesicles, containing various molecules such as proteins, nucleic acids, and lipids reflect the physiological state of their parent cells. Analyzing the cargo within exosomes, could provide valuable insights into the presence of specific biomarkers associated with diseases such as cancer,^30^ neurodegenerative disorders,^31^ and cardiovascular diseases.^32^ In addition to their diagnostic potential, exosomes also hold significant therapeutic value. These natural carriers can be engineered to deliver therapeutic agents, such as drugs or nucleic acids, to specific target cells or tissues. The unique properties of exosomes, including their stability, biocompatibility, and ability to cross biological barriers, make them attractive vehicles for targeted drug delivery and gene therapy.^33^ Furthermore, exosomes can mediate intercellular communication and modulate cellular functions, presenting opportunities for developing novel therapeutic strategies.^34^, ^35^

In the following sections, we discuss the role of exosomes derived from various sources in the pathogenesis of asthma. Furthermore, we explore the potential of harnessing exosomes as therapeutic tools for the treatment of asthma. By exploring the multifaceted nature of exosomes and their potential applications, we aim to uncover new avenues for managing and alleviating the symptoms of asthma.

### 2.2 MVs

MVs are a type of EVs that are released from the cell membrane by outward budding or blebbing of the plasma membrane. MVs range in size from 100 nm to 1000 nm in diameter, larger than exosomes (30–100 nm).^36^ They are formed by outward protrusion and budding of the plasma membrane, initiated by an increase in intracellular calcium that activates enzymes like calpain and gelsolin to remodel the cytoskeleton.^37^ MVs contain proteins, mRNA, miRNA, lipids and can transfer this cargo between cells, facilitating intercellular communication.^38^ They play roles in physiological processes like coagulation, inflammation, stem cell maintenance, and pathological processes like cancer progression, metastasis, and chemoresistance.^39^ MVs are released from various cell types including platelets, immune cells, tumor cells, placental cells, and stem cells. Their release can be constitutive or induced by cellular activation, stress, or apoptosis.

### 2.3 Apoptotic body

Apoptotic bodies (ApoBDs) are a type of extracellular vesicle formed during the final stages of apoptosis, or programmed cell death. ApoBDs contain fragmented nuclear material (chromatin bodies), cytoplasmic contents, and organelles from the dying cell.^40^ Their surface exposes phosphatidylserine, acting as an “eat me” signal for phagocytic clearance. ApoBDs are rapidly phagocytosed by neighboring cells or macrophages, facilitating efficient clearance of apoptotic cells without inducing inflammation. They can transfer biomolecular cargo like proteins, RNA, and DNA to recipient cells. ApoBDs are involved in embryonic development, immune regulation, and pathological conditions like cancer progression.^41^, ^42^

## 3 CELL‐DERIVED EXOSOMES IN PATHOGENESIS OF ASTHMA

### 3.1 Macrophage

**Figure 1:**
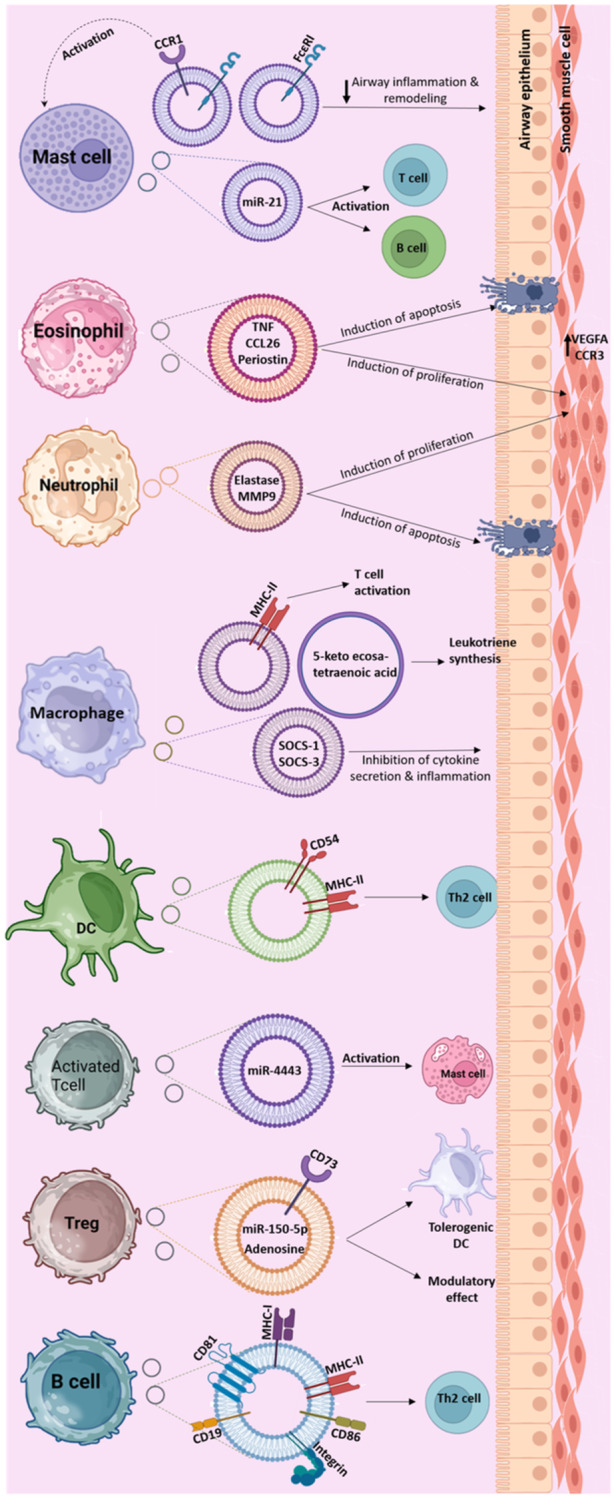
Role of exosomes from different cellular sources in the pathogenesis of asthma. Most of the immune cells secrete exosome with common and distinct contents. The figure shows a summary of the main reported roles of exosomes in the pathogenesis of asthma discussed in this review in detail. MMP9, matrix metallopeptidase 9; SOCS, suppressor of cytokine signaling; VEGFA, vascular endothelial growth factor A.

Macrophages and monocytes also are involved in asthmatic inflammation. Several studies have demonstrated the pro‐inflammatory role of M1 macrophages and the anti‐inflammatory role of M2 macrophages in asthma.^43^, ^44^ Macrophages are also major sources of pulmonary EVs. Such EVs containing MHC‐II and costimulatory molecules serve as an alternative pathway for antigen presentation in the lung (Figure 1).^45^ It has been shown that EVs derived from M2‐like alveolar macrophages contain cytokine signaling‐I (SOCS‐I) and SOCS‐3 which inhibits JAK–STAT inflammatory signaling‌ and cytokine secretion in epithelial cells.^46^, ^47^ Therefore, the dysregulated cytokine production in chronic inflammatory airway diseases such as asthma might be at least in part, due to impaired EV‐mediated trans‐cellular delivery of these proteins. Moreover, alveolar macrophages produce EVs containing pathogen‐derived pro‐inflammatory molecules upon infection with mycobacteria. These EVs stimulate tumor necrosis factor‐α (TNF‐α) secretion through toll‐like receptor (TLR)‐mediated NF‐κB signaling by macrophages and neutrophils.^48^, ^49^, ^50^ EVs carrying biologically active TNF‐α have also been reported to be released by macrophages following lipopolysaccharides (LPS) stimulation.^51^ Air pollutions including particulate matter have been associated with childhood asthma and wheezing.^52^ In vitro exposure of macrophages to particulate matter stimulated secretion of EVs which in turn, induced secretion of pro‐inflammatory cytokine such as TNF‐α and IL‐6 in pulmonary epithelial cells.^53^ Macrophages could also release exosomes which are reported to be involved in in Type 2 immune responses. Exosomes from monocyte‐derived macrophages contain 5‐keto eicosatetraenoic acid and enzymes involved in leukotriene (LT) synthesis, particularly LTB4, which contributes to the allergic inflammation and remodeling changes of asthmatic airways.^54^ Moreover, it has been shown that M2 macrophages can promote the differentiation of innate lymphoid cell (ILC)2, key elements of the innate type 2 immune responses, probably via secretion of exosomes.^55^

### 3.2 Dendritic cell

Dendritic cells (DCs) as primary APCs in the immune system are key players in immune defense, surveillance, and homeostasis through activation of T cell responses.^56^ DCs also contribute to the pathogenesis of asthma as increased numbers of pulmonary DCs have been reported upon exposure to environmental allergens.^57^ In fact, the interaction between airway epithelial cells and DCs through production of alarmins contributes to the induction of type 2 immune responses in asthmatics.^58^, ^59^ DCs secrete exosomes with diverse properties based on the activation nature and the type of cytokines present in the environment.^60^ DC‐derived exosomes carry costimulatory molecules and Ag‐MHC‐II complexes along with CD54, an adhesion molecule that enables their interaction with lymphocyte function‐associated antigen‐1 (LFA‐1) on T cells and subsequent activation of these cells.^61^ Studies have shown that DC‐derived exosomes might serve as an alternative route for presentation of aeroallergens to T cells, thus promoting allergic inflammation via stimulating type 2 cytokines production.^61^, ^62^ A recent study reported that exosomes derived from DCs activated with thymic stromal lymphopoietin (TSLP), an epithelial alarmin, express OX40 ligand (OX40L) on their surface which induces proliferation of CD4^+^ T cells and their differentiation of towards a Th2 phenotype in vitro.^63^ DC‐derived EVs containing enzymes for biosynthesis of LTs, key elements in airway remodeling, and chemotactic eicosanoids, involved in granulocyte migration, contribute to pathogenesis of asthma.^54^ However, the majority of the studies on DC‐derived EVs in the context of asthma had been performed using ex vivo generated bone marrow‐ or monocyte‐derived DCs. Given the heterogeneous nature of pulmonary DCs, additional investigations are necessary to elucidate the involvement of exosomes derived from distinct subsets of lung DCs, including conventional types 1 and 2 DCs, as well as plasmacytoid DCs, in the pathogenesis of asthma.

### 3.3 Eosinophils

Eosinophils are innate immune cells that have long been recognized as one of the characteristic features of asthma pathophysiology.^64^ They are elevated in asthmatic airways causing airway inflammation via production of a variety of enzymes and mediators including MBP, EPO, ECP, metalloproteinases, LTs as well as reactive oxygen species (ROS) or NO.^64^, ^65^ It has been shown that eosinophils can secrete EVs in response to IFN‐γ stimulation and the level of EV secretion and their capacities differ between asthmatic patients and healthy subjects.^66^ Asthmatic patients produce higher levels of EVs and such EVs containing eosinophil‐derived enzymes such as MBP, EPO and EPO may influence asthmatic airways and several lung‐resident cells.^66^ Exosomes derived from asthmatics promote eosinophil chemotaxis via upregulation of adhesion molecules such as ICAM‐1 and integrin α2.^67^ Moreover, eosinophil‐derived exosomes from asthmatic patients contribute to asthma remodeling by excreting a variety of pro‐inflammatory effects in lung structural cells. They induce apoptosis in airway epithelial cells impeding their wound healing capacity along with increased expression of several cytokines such as CCL26, TNF and periostin.^68^ Such exosomes also induce the proliferation of bronchial smooth muscle cells through ERK1/2 phosphorylation pathway and upregulate the gene expression of CCR3 and VEGFA in these cells.^68^

### 3.4 Mast cells

Mast cells like many other cell types secrete exosomes containing functional mRNA and microRNAs, MHCII molecules, costimulatory molecules, integrins and a variety of proteins common to all exosomes such as tetraspanins, chaperones, and cytoskeletal proteins.^69^, ^70^, ^71^, ^72^ The contribution of MC‐derived exosomes in pathophysiology of asthma and the lung inflammatory response has been the subject of several studies. It has been shown that exosomes from bone marrow‐derived mast cells (BMMCs) are capable of inducing activation of T cells and B cells in a contact‐independent manner, suggesting a role for MC‐derived mast cells in the recruitment of B and T cells to the lungs.^71^ On the other hand, exosomes derived from BMMCs, carrying high‐affinity IgE receptor (FcεRI) show anti‐IgE effect which results in modulating airway inflammation and remodeling in chronic asthma.^73^ It has also been shown that, costimulation of FcεRI and CC chemokine receptor1 (CCR1) on the surface of mast cells results in greater mast cells degranulation.^74^ In line with this finding, mast cell‐derived exosomes upregulating CCR1 may contribute to enhancement of mast cell activation through transfer of CCR1 to other mast cells.^75^ Moreover, inhibition of miR‐21 expression in MC‐derived exosomes contributes to regulation of oxidative stress and inflammatory response in asthmatic airway epithelial cells.^76^

### 3.5 Neutrophils

The role of neutrophils in airway inflammation and remodeling has been shown especially in cases with severe asthma and poor response to treatment.^77^, ^78^ Neutrophils contribute to asthma pathogenesis and development by secretion of inflammatory mediators such as cytokines (e.g., IL‐8, IL‐6, IL9, IL‐12, and IFN‐γ), enzymes (e.g., elastase), ROS and release of extracellular traps.^79^ Evidence for involvement of neutrophil‐derived exosomes in asthma pathogenesis and airway remodeling was revealed using exosomes isolated from LPS‐treated neutrophils which had the capacity to modulate apoptosis and proliferation of airway smooth muscle cells.^80^ Such effect might be partly due to the function of lncRNA CRNDE in neutrophil derived exosomes, as in vivo knock down of the CRNDE gene in neutrophils that significantly reduced the thickness of bronchial smooth muscle in asthmatic mice.^81^ Another mechanism for involvement of neutrophil‐derived exosomes in epithelial damage might be through high level of enzymatically active matrix metalloproteinase 9 (MMP‐9) affecting the integrity of epithelial cells.^82^ The proteolytic activity of exosomal neutrophil elastase on extracellular matrix (ECM) has been also proposed as a mechanism of airway remodeling in neutrophil‐driven inflammatory lung diseases.^83^

### 3.6 Lymphocytes

T and B lymphocytes as the main mediators of adaptive immune responses are significantly implicated in pathogenesis of bronchial asthma through cytokine production, secretion of IgE specific for allergens as well as production of agents that exert inflammatory changes in other cell types involved in pathogenesis of asthma.^84^ Activation of mast cells by T cell‐derived EVs, carrying mast cell‐activating factors such as miR‐4443, has been proposed as an alternative pathway for mast cell activation at distant inflammatory sites which involves the MAPK signaling pathway.^85^, ^86^ Accordingly, EVs from activated T cells induced ERK kinase phosphorylation in recipient immune cells due to upregulation of RAS/MAPK signaling pathway proteins supporting a mechanistic role of T cell‐derived exosomes in cellular activation.^87^ On the other hand, characterization of EVs obtained from Treg cells revealed a modulatory capacity for these EVs due to the presence of miR‐150‐5p and miR‐142‐3p, as interaction with Treg‐derived EVs induced a tolerogenic phenotype in DCs following LPS stimulation.^88^ In another in vitro study, the modulatory effect of Treg‐derived EVs was attributed to the expression of CD73 and production of adenosine,^89^ suggesting new mechanisms for regulatory functions of Tregs in immunological processes. It has been demonstrated that B cells secrete exosomes carrying antigen presenting molecules (MHC‐I and ‐II), costimulatory molecules (CD86), integrins (β1 and β2) as well as subunits of the coreceptor complex (CD19, CD81).^90^, ^91^ Such exosomes when loaded with peptides had the capacity to potentiate antigen presentation to T cells, inducing T cell proliferation and differentiation to IL‐5 and IL‐13 producing Th2 cells,^91^ highlighting the potential role of B cell‐derived exosomes in allergic diseases. However, none of these studies was performed on the functional effect of T‐cell‐ or B cell‐derived EVs on lung cells specifically or in the context of bronchial asthma.

### 3.7 Bronchial epithelial cells

The airway epithelium acts as the primary defense against environmental threats and regulates underlying immunity, playing a crucial role in both host defense and the development of airway inflammatory diseases like asthma.^92^ Besides, bronchial epithelial cells as the major source of exosomes in asthmatic lungs can affect the neighboring epithelial and immune cells via cell‐to‐cell communication.^93^ in vitro and in vivo studies show that airway epithelial cells under the stress of bronchoconstriction which is a major hallmark of bronchial asthma, secrete tissue factor‐bearing exosomes which promotes angiogenesis and pulmonary fibrosis in asthmatic lungs.^94^ Moreover, in asthmatic inflammation epithelial cells undergo enhanced exosome secretion under the influence of IL‐13 which in turn, induces proliferation and chemotaxis of undifferentiated macrophages into the lungs.^93^ Accordingly, use of GW4869, an inhibitor of exosome release, reduced pulmonary inflammation via reduction in proliferating population of monocytes.^93^ The exchange of exosomes between airway epithelial cells results in qualitative and quantitative alterations in airway secretions associated with altered mucin and miRNA content of the epithelia‐derived exosomes.^95^ It has been shown that decreased content of three microRNA (miRNAs) including miR‐92b, miR‐210, and miR‐34a, all of which are involve in modulation of immune responses, in IL‐13‐treated human bronchial epithelial cells is associated with airway obstruction.^96^ Such information on differential expression of molecules in exosomes provide us useful tools to design and develop new strategies for treatment of corticosteroid refractory asthma.

## 4 BIOFLUIDS‐DERIVED EXOSOMES IN ASTHMA

In the context of asthma, not only cellular component and its inflammatory mediators are important, but also the soluble inflammatory factors in biofluids also play a crucial role in the pathogenesis and development asthma. Exosomes derived from biofluids in asthma are released by various immune and structural cells and identified in bronchoalveolar lavage fluid (BALF), serum, plasma, and nasopharynx lavage fluid (NLF). These exosomes carry different molecules such as proteins, lipid, cholesterol and nucleic acid that can modulate immune responses and inflammation in asthma. They also contain high level of certain mediators, such as leukotriene biosynthetic enzymes and miRNAs that are involved in regulating inflammation, oxidative stress, and vascular function in asthma. Exosomes derived biofluids in asthma serve as potential biomarkers for asthma diagnosis and severity, as they reflect the cellular and molecular status of the donor cells. This review assessed the various component carried by BALF, NLF, and blood exosomes and explained the potential roles of these components in the formation and development of asthma and their application in asthma diagnosis.

### 4.1 BALF

The initial report on the presence of exosomes in the BALF of humans was documented by Admyre et al. in 2003.^97^ Exosomes and their components are considered as important part of BALF in asthma. Exosomes from BALF present antigens to the adaptive immune system and stimulate the activation of alveolar macrophages, mast cells and eosinophils in the lung, leading to airway changes, increased airway sensitivity, and obstruction in asthma.^98^ Prado et al. demonstrated that intranasal administration of exosomes derived from the BALF of tolerized mice can effectively prevent allergic sensitization.^99^ These tolerogenic exosomes effectively restrict the IgE response and suppress Th2 cytokine production, thereby mitigating airway inflammation. This beneficial effect is associated with an upregulation of regulatory cytokines, such as transforming growth factor‐β (TGF‐β). Moreover, studies have demonstrated that exosomes derived from BALF could potentially contribute to subclinical inflammation in asthmatic individuals by enhancing the production of cytokines and leukotriene C4 (LTC4) in the airway epithelium.^100^ They additionally discovered that a fraction of these exosomes can be traced back to bronchial epithelial cells, as evidenced by their expression of the epithelial marker mucin‐1 in conjunction with human leukocyte antigen DR. It has been shown that epithelial cells and macrophages are main source of exosomes in BALF of asthmatic lungs.^93^, ^101^ Furthermore, exosomes derived from bronchial epithelial cells treated with IL‐13 could effectively induce the proliferation of undifferentiated macrophages in individuals with asthma.^93^

Differential expression of miRNAs from exosomes in the BALF of asthmatics can be used as potential biomarker for asthma diagnosis and severity. Levänen et al.,^102^ for the first time, detected significant disparities in the expression of 24 BALF exosomal miRNAs, which encompassed members from the let‐7 and miRNA‐200 families, effectively distinguishing between individuals without asthma and those diagnosed with the condition.^102^ These alterations could potentially play a crucial role in driving the inflammatory response that culminates in bronchial hyper responsiveness and the development of asthma. Francisco‐Garcia et al. found that exosomes from severe asthmatic patients had different microRNA content that influenced cellular pathways related to airway inflammation and remodeling. They also showed that this microRNA content was associated with Forced expiratory volume1 (FEV1) and the levels of eosinophils and neutrophils in the airways.^103^ Subsequent investigation revealed a diminished expression of miR‐493‐5p in both lung tissue and BALF of mice with asthma.^104^ MiR‐493‐5p was found to exert a negative regulatory effect on the differentiation of Th9 cells by specifically targeting FOXO1. Thus, DC‐derived exosomal miR‐493‐5p/FOXO1/Th9 may potentially serve as a therapeutic target in the development of asthma.

### 4.2 NLF

NLF is a fluid that contains saline and nasal secretions, which include epithelial cells, neutrophils, eosinophils, lymphocytes and their released substances.^105^ NLF reflects the inflammatory state of the nasal cavities, which are a specific part of the upper airways. Rhinitis has been the main focus of nasal fluid studies, as it causes inflammation and excessive secretion of the nasal mucosa.^105^, ^106^ Zhou et al. investigated the effects of exosomes from epithelial cells in the nasal fluid of patients with chronic rhinosinusitis with nasal polyp (CRSwNP) with or without asthma.^107^ They discovered that these exosomes contained proteins that could stimulate cell growth pathways like p53, which could contribute to the changes in the nasal mucosa. Another study in this field analyzed the proteome of exosomes derived from nasal lavage fluid to investigate the potential influence of nasal exosomes on inflammatory response in asthma.^108^ The study showed that some exosomal proteins that are involved in barrier and antimicrobial functions, such as filaggrin, hornerin and three immunoglobulin‐related proteins, were lower in asthmatic patients than in healthy ones. The study also suggested that nasal exosomal proteins could attract innate immune cells to the airway epithelium, which is the first layer of protection against pathogens and allergens. Therefore, the decreased levels of these exosomal proteins in individuals with airway diseases may render them more susceptible to infections, potentially leading to significant clinical consequences in terms of disease progression.

### 4.3 Serum and plasma

Serum or plasma is another main source of exosomes that are involved in asthma pathology. MiR‐21 is a key serum exosome miRNA that is associated with asthma pathogenesis and severity. Numerous studies have suggested its potential as noninvasive biomarkers for identifying the phenotype, endotype, and severity of asthma.^109^, ^110^, ^111^, ^112^ Rostami et al. reported that miR‐21 could diagnose moderate asthma with 76% accuracy.^111^ MiR‐21 could also be viewed as a promising potential biomarker for assessing the response to asthma therapy in children with asthma, as demonstrated in the manuscript by Elbehidy and colleaguest^112^ Other studies reported a correlation between miR‐21 and immunological parameters involved in Th2 responses. One study found a positive association between the levels of miR‐21 and IL‐4 in the blood, which confirmed the involvement of this miRNA in activating Th2 cells and causing asthma.^113^ Serum levels of miRNA‐21 were inversely related to serum IL‐12p35 levels and FEV1, while it was directly related to the number of eosinophils in sputum and blood.^112^

Several studies have presented a plasma/serum exosomal miRNA profile as a valuable biomarker for diagnosing and distinguishing clinical phenotypes of asthma, exhibiting favorable sensitivity, specificity, as well as positive and negative predictive values. The study by Atashbasteh et al. suggested that a combination of increased miR‐125b and decreased miR‐124, miR‐133b, and miR‐130a expression levels could be a useful biomarker for asthma diagnosis.^114^ Rodrigo‐Muñoz et al. also investigated the miRNA profile of eosinophils and identified three miRNAs (miR‐185‐5p, miR‐144‐5p, and miR‐1246) that could distinguish asthma patients from healthy individuals using a logistic regression model.^22^ Vázquez‐Mera et al. discovered a composition of T‐cell miRNAs, namely miR‐21‐5p, miR‐126‐3p, miR‐146a‐5p, and miR‐215‐5p, released into the serum within exosomes. These miRNAs were identified as clinically relevant noninvasive biomarkers for assessing the phenotype and severity of asthma.^109^ Suzuki et al. used serum exosomal RNA to discriminate varied types of asthma.^115^ The analysis of microRNA expression showed that four miRNAs (miR‐128, miR‐140‐3p, miR‐196b‐5p, and miR‐486‐5p) were more abundant in severe asthma patients than in mild‐to‐moderate asthma patients and healthy volunteers. These miRNAs are involved in the regulation of biological pathways related to cell signaling, adhesion, and survival. High level of serum exosomes miR‐21‐5p, miR‐126‐3p, miR146a‐5p and miR‐215‐5p were reported in moderate to severe patients.^110^

## 5 THE THERAPEUTIC POTENTIAL OF EXOSOMES

Owing to their inherent targeting ability, low immunogenicity, innate stability, modification flexibility, and excellent tissue/cell permeabilization capacity, exosomes hold promise as ideal candidates for designing more exciting drug and vaccine delivery systems compared to other nanoparticulate drug delivery systems, such as liposomes.^19^, ^116^ Different molecular cargos including small molecules, proteins, and nucleic acids can be delivered by exosomes to specific recipient tissues. For example, exosomes loaded with curcumin, a plant‐derived small molecule with immunomodulatory and anticancer effects, have been shown to increase the solubility, stability, and bioavailability of the curcumin.^117^ As natural intercellular transporters of RNAs, exosomes could potentially be considered as new candidates for therapeutic delivery of small RNAs, for example, pre‐miRNAs and miRNAs. In a study by Ohno et al.,^118^ showed that targeted delivery of exosomes expressing exogenous let‐7a miRNA, an important tumor suppressor gene, to EGFR expressing breast cancer cells in RAG2(‐/‐) mice results in strong suppression of tumor growth. In addition, exosomes derived from ex vivo‐manipulated donor cells have been used in several studies. For example, DCs adenovirally transduced with IDO secrete EVs with anti‐inflammatory effects which are able to reverse established arthritis in animal models.^119^ There are also increasing evidence for the possibility of using exosomes as promising treatment alternatives for inflammatory conditions such as chronic respiratory diseases, for example, COPD and asthma. In multiple preclinical studies the therapeutic potential of mesenchymal stem cells (MSC)‐derived exosomes in inflammatory lung diseases through inhibition of lung inflammation and vascular remodeling has been shown.^120^, ^121^ The potential use of exosomes as “mucosal vaccines” has also been suggested since the administration of BALF‐derived exosomes to allergen‐immunized mice before immunization led to protection of naïve mice from allergen‐induced allergic response.^122^

## 6 USE OF EXOSOMES AS IMMUNOTHERAPEUTIC TOOLS IN ASTHMA

Thanks to the advances in drug therapy centered on inhaled corticosteroids (ICS), many patients with persistent asthma can be controlled effectively. However, such “symptomatic treatments” do not specifically target clinical characteristics of asthma.^123^, ^124^ Allergen immunotherapy is a potentially disease‐modifying therapy which targets allergen‐specific type 2 immune responses and aims to achieve reciprocal regulation of effector and Treg cell subsets.^125^, ^126^, ^127^, ^128^ Direct targeting of Th2‐type cytokines (e.g. IL‐4, IL‐5, IL‐9, IL‐13, IL‐23, IL‐25, IL‐33) or IgE antibody are other promising approaches of immunotherapy.^129^, ^130^, ^131^ Recent studies in this area have provided insight into the immunotherapeutic application of exosomes in the context of asthma since exosomes have potential to boost, deviate, or suppress immune responses.^73^ Generation of tolerance mainly via allergen nonresponsive Treg cells is the ideal goal of allergy immunotherapy. The results of previous studies suggest that exosomes might also be involved in the induction of tolerance. Exosomes derived from MSCs have shown promising potential in inducing immune tolerance in asthma. The modulatory effect of MSC‐derived exosomes on the immune responses through expansion of Treg cells has been shown on different studies.^132^, ^133^ MSCs are self‐renewing multipotent stromal cells that possess the ability to differentiate into various cell types, including osteoblasts, chondrocytes, adipocytes, and myocytes.^134^ Due to their remarkable capacity to differentiate into specific cell types, anti‐inflammatory and immune‐modulatory properties, MSCs are a promising resource for cell‐based therapy of inflammatory diseases.^135^ Studies investigating the effect of MSC‐derived exosomes on asthma pathogenesis and airway remodeling revealed an upregulation in IL‐10 and TGF‐β resulting in increased proliferation and immunosuppressive capacity of Treg cells, ameliorating airway inflammation of asthmatic mice.^136^ Interestingly, the coculture of bone marrow‐derived MSCs with PBMCs of asthmatic patients resulted in enhanced secretion of IL‐10 and TGF‐β by PBMCs.^136^ Another study revealed that systemic administration of EVs from human adipose tissue‐derived MSCs reduced accumulation of CD3^+^CD4^+^ T cells in BALF and lungs and inhibited accumulation of eosinophils in lungs along with modulation of airway remodeling.^137^ These findings highlight the potential of MSC‐derived exosomes as a therapeutic tool to induce immune tolerance and mitigate allergic airway inflammation in asthma. The potential of MSC‐derived exosomes to induce tolerogenic DCs^138^ and to suppress Th2 differentiation^139^, ^140^, ^141^ is another mechanism that could be taken into account for designing immunotherapeutic strategies for allergy and asthma. Moreover, exosomes carrying specific antigen and MHC‐II called “tolerosomes” could be isolated from serum of ovalbumin (OVA)‐fed mice.^142^ Such exosomes originating from intestinal epithelial cells are capable of inducing tolerance when transferred into naïve recipients.^143^ Induction of Th1 responses and thereby enhanced IgG production using exosomes has also been demonstrated in previous studies.^62^, ^144^, ^145^

IgE‐antigen complexes preferentially bind to CD23, the low affinity IgE receptor on B cells, which facilitates delivering antigen to dendritic cells and antigen presentation to T cells via a process called IgE‐facilitated antigen presentation. It has been shown that B cell‐derived exosomes carrying CD23, IgE and MHC‐II when exposed to IgE‐immune complexes increase specific IgG production and reduce IgE serum level on the other hand.^146^, ^147^ Moreover, as indicated earlier, expression of high‐affinity IgE receptor, FcεRI, on the surface of MC‐derived exosomes may contribute to IgE neutralization and thereby alleviates allergic symptoms.^73^ Therefore, B cell‐derived exosomes or exosomes from different sources carrying allergens or p‐MHC complexes could be engineered to express FcεRI or other molecules such as costimulatory molecules to be utilized as immunotherapeutic agents to reduce systemic IgE levels in asthma patients. Such engineered exosomes could be further modified to be used as a lyophilized dry powder to enhance their stability and their inhaled biodistribution in the lung.^148^ Table 1 has summarized different proposed mechanisms through which the exosomes might be used to modulate immune responses in asthmatics.

**Table 1:** Exosomes as therapeutic tools in asthma.

Strategy	Mechanism	Source of exosome	Reference
Induction of tolerance	Induction of anti‐inflammatory cytokines	Human bone marrow‐derived MSCs	[136]
	Induction of tolerogenic DCs	Murine adipose‐derived MSCs	[138]
	Reduced accumulation of inflammatory cells	Human adipose‐derived MSCs	[137]
Immune deviation	Suppression of Th2 differentiation	Human bone marrow‐derived MSCs	[139]
		Human adipose‐derived MSCs	[141]
	Induction of Th1 differentiation	OVA‐pulsed murine DCs	[62]
		LPS‐stimulated human monocytic cell line	[144]
		Diphtheria toxoid‐pulsed murine DCs	[145]
Interfering with IgE‐mediated response	Reduction of IgE serum level	Murine B‐cell derived exosomes	[147]
	IgE neutralization	Murine bone marrow‐derived MCs	[73]

*Note*: Exosomes have been proposed as therapeutic tools in different studies. Exosomes originated from different sources have shown promising potential to target clinical characteristics of allergic diseases including asthma through different mechanisms. MSC: mesenchymal stem cells; OVA: Ovalbumin; DC: Dendritic cell; LPS: Lipopolysaccharide; MC: Mast cell

## 7 CONCLUSION

Exosomes having the capacity to transport proteins, RNAs and other bioactive substances, play a crucial role in intercellular communication and are of particular interest in scientific research in the field of physiological processes and multiple diseases. Bronchial asthma is a complex, multifactorial disease leading to structural changes of the airways reflecting a complex interplay between epithelial barrier dysfunction and dysregulated immune cell function. An increasing number of reports have highlighted a role for exosomes in the pathology of asthma, COPD and other inflammatory diseases. It has been shown that almost all types of immune cells as well as pulmonary epithelial cells release exosomes. In asthmatics, alterations in exosomal content have also been described in bodily fluids such as BALF, NLF and serum. Numerous studies have described the involvement of these exosomes in various aspects of immune activation, modulation, and surveillance. Furthermore, the differential exosomal content in physiological and pathological states has driven their promise as valuable diagnostic biomarkers for asthma phenotype and severity. Moreover, exosomes have great potential in designing immunotherapeutic methods due to their capacity to suppress or to promote immune responses. The use of exosomes derived from MSCs with immunomodulatory properties as well as Treg and tolerogenic DC‐derived exosomes has been suggested for immunotherapy of allergy. Exosomes carrying allergens or p‐MHC complexes can also be engineered to express costimulatory molecules or FcεRI to be utilized as immunotherapeutic agents for treatment of asthma. However, despite significant efforts in this relatively new research area, further fundamental studies on exosomes and their role in the pathogenesis of asthma are necessary. These studies are crucial to advance our understanding and explore the potential applications of exosomes for the development of therapeutic tools in the field of asthma.

## AUTHOR CONTRIBUTIONS

**Zahra Kanannejad**: Investigation; writing—original draft; writing—review and editing. **Samaneh Arab**: Investigation; writing—original draft. **Saeede Soleimanian**: Investigation; Writing—original draft. **Amirhossein Mazare**: Investigation; writing—original draft. **Nasim Kheshtchin**: Investigation; supervision; writing—original draft; writing—review and editing.

## CONFLICT OF INTEREST STATEMENT

The authors declare no conflicts of interest.
